# Bidirectional *in vivo* structural dendritic spine plasticity revealed by two-photon glutamate uncaging in the mouse neocortex

**DOI:** 10.1038/s41598-019-50445-0

**Published:** 2019-09-26

**Authors:** Jun Noguchi, Akira Nagaoka, Tatsuya Hayama, Hasan Ucar, Sho Yagishita, Noriko Takahashi, Haruo Kasai

**Affiliations:** 1 Department of Ultrastructural Research, National Institute of Neuroscience, National Center of Neurology and Psychiatry, Kodaira, Tokyo 187-8502 Japan; 2 Laboratory of Structural Physiology, Center for Disease Biology and Integrative Medicine, Faculty of Medicine, The University of Tokyo, Bunkyo-ku, Tokyo 113-0033 Japan; 3 International Research Center for Neurointelligence (WPI-IRCN), UTIAS, The University of Tokyo, Bunkyo-ku, Tokyo Japan; 4 Department of Physiology, Kitasato University School of Medicine, Sagamihara, Kanagawa 252-0374 Japan

**Keywords:** Cortex, Long-term potentiation

## Abstract

Most excitatory synapses in the brain form on dendritic spines. Two-photon uncaging of glutamate is widely utilized to characterize the structural plasticity of dendritic spines in brain slice preparations *in vitro*. In the present study, glutamate uncaging was used to investigate spine plasticity, for the first time, *in vivo*. A caged glutamate compound was applied to the surface of the mouse visual cortex *in vivo*, revealing the successful induction of spine enlargement by repetitive two-photon uncaging in a magnesium free solution. Notably, this induction occurred in a smaller fraction of spines in the neocortex *in vivo* (22%) than in hippocampal slices (95%). Once induced, the time course and mean long-term enlargement amplitudes were similar to those found in hippocampal slices. However, low-frequency (1–2 Hz) glutamate uncaging in the presence of magnesium caused spine shrinkage in a similar fraction (35%) of spines as in hippocampal slices, though spread to neighboring spines occurred less frequently than it did in hippocampal slices. Thus, the structural plasticity may occur similarly in the neocortex *in vivo* as in hippocampal slices, although it happened less frequently in our experimental conditions.

## Introduction

Most excitatory synapses in the brain form on dendritic spines. The volume of dendritic spines is tightly correlated with the functional expression of glutamate receptors in the young hippocampal slice preparations^1–6^ and in the adult mouse neocortex *in vivo*^7^. Spine volume changes accompany long-term potentiation and depression of synapses in hippocampal slices^8–12^. Such volume changes may lead to the generation and elimination of spines^13–19^ as well as impaired structural plasticity which ultimately leads to pathological neuronal circuitry^18,20,21^.

Two-photon uncaging of caged glutamate compounds is the only method that reliably stimulates single spines^1^. Furthermore, it is widely used to characterize structural spine changes *in vitro*. Spine enlargement is most robustly induced by uncaging glutamate in the absence of external magnesium (Mg^2+^), such that *N*-methyl-d-aspartic acid (NMDA) receptors are maximally activated^8,10,22–26^. Spine shrinkage is induced by low-frequency uncaging^11,12,27^. However, assessing spine plasticity with two-photon uncaging has never been applied *in vivo* because of the technical difficulties associated with uncaging in a living animal. The characteristics of structural plasticity *in vivo* are therefore unknown in the adult mouse neocortex.

We previously established a glutamate uncaging method *in vivo* in which a caged glutamate compound was applied to the surface of the brain. This allowed the compound to spread to the superficial extracellular space of the neocortex by passive diffusion^7^. The present study extends this work to assess the structural plasticity of dendritic spines, for the first time, *in vivo*.

## Results

### *In vivo* spine enlargement

**Figure 1:**
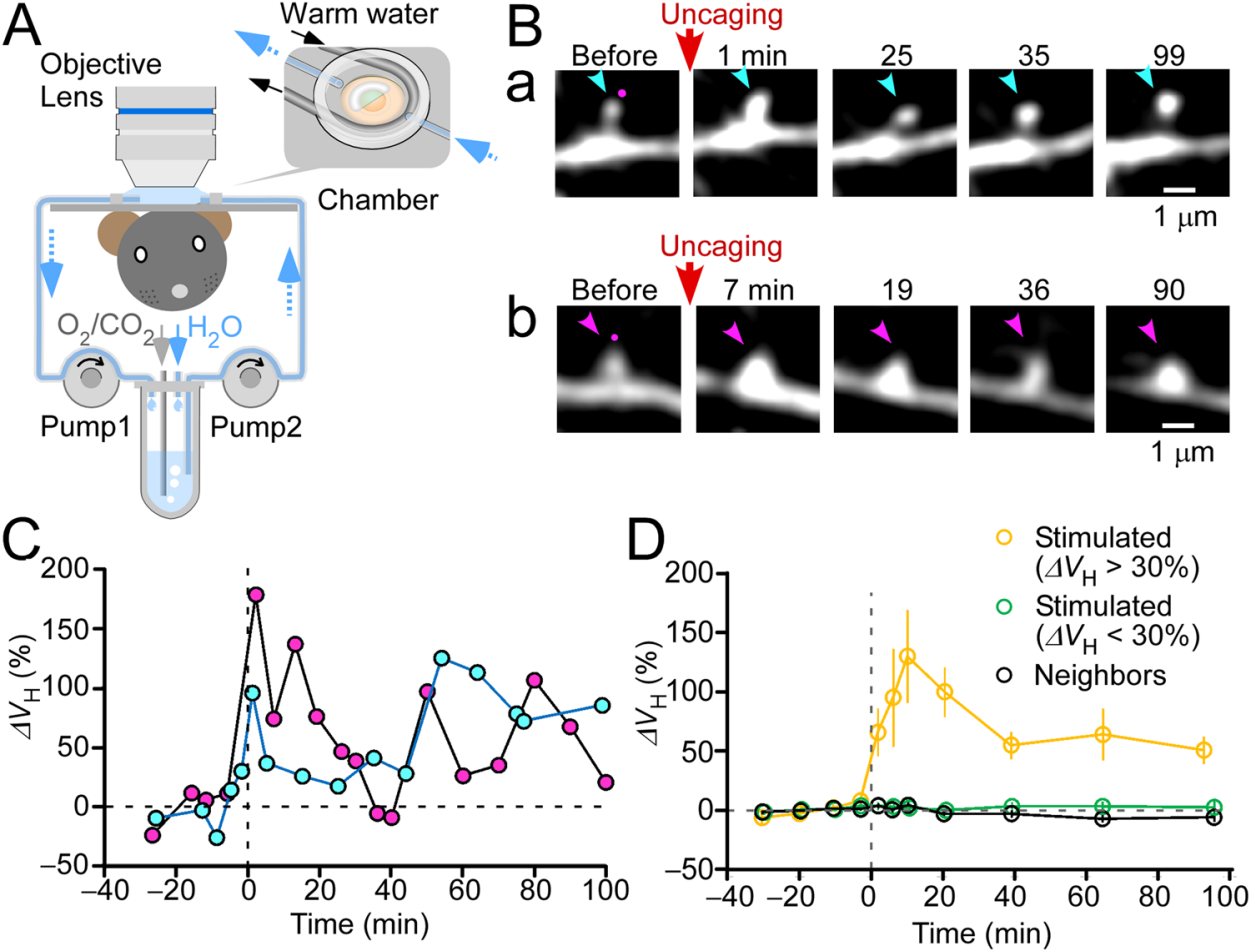
Induction of spine enlargement in the visual cortex *in vivo*. (**A**) Schematic of the experimental design. Transgenic mice expressing yellow fluorescent protein (YFP) or green fluorescent protein (GFP) in neocortex layer 5/6 pyramidal neurons were urethane-anesthetized and placed under an objective lens using a metal frame. Skull and dura over the V1 neocortical area were carefully removed, and a half-moon-shaped coverslip was placed on the brain surface. A perfusion solution containing caged glutamate and 10 µM tetrodotoxin (TTX), but no magnesium (Mg^2+^), was steadily circulated using peristaltic pumps. After diffusing the caged glutamate into the brain parenchyma, caged glutamate was photolyzed at the tip of dendritic spines via two-photon uncaging at 720 nm. Dendrite images were obtained in another channel (see the “Methods” section for details). (**B**) Time-lapse images of the stimulated spines. Several spines (4.6 spines on average) per dendrite were stimulated via repetitive two-photon glutamate uncaging. Magenta dots designate uncaging cites. Cyan and magenta arrowheads indicate stimulated spines. (**C**) Time course for spine a (cyan) and spine b (magenta) volume increases in panel (B). (**D**) The averaged time courses of spine-head volume increments for spines with >30% enlargement (orange circles), spines with <30% enlargement (green circles), and unstimulated neighboring spines (black circles) (*n* = 16, *n* = 58, and *n* = 92 for enlarged, unenlarged, and neighboring spines, respectively).

A compound for two-photon uncaging of glutamate (Methods) was applied to single spines of the tuft dendrites of layer 5/6 pyramidal neurons in the visual cortex of adult mice (*n* = 18) *in vivo*^7^. A yellow fluorescent protein (YFP)-expressing mouse line (H) or green fluorescent protein (GFP)-expressing mouse line (M), in which a subset of pyramidal neurons are labelled in a layer 5/6 selective manner, were used. Mice were anesthetized with urethane and xylazine and placed under a microscope objective lens using an imaging chamber that was firmly attached to the mouse skull (Fig. 1A). To activate NMDA receptors effectively, the recording chamber was superfused with artificial cerebrospinal fluid containing no magnesium (Mg^2+^) ions. Caged glutamate was thereafter superfused (Fig. 1A, Supplementary Fig. 1A). Spine head volume (*V*_H_) fluctuations before uncaging were quantified as coefficients of variation (CVs) (Supplementary Fig. 1B). The CV of *in vivo* neocortex spines was 15% ± 16% (mean ± standard deviation [SD]; 227 spines), compared to 21% in hippocampal slices^8^, demonstrating a similar stability of our recording conditions as in slices.

**Figure 2:**
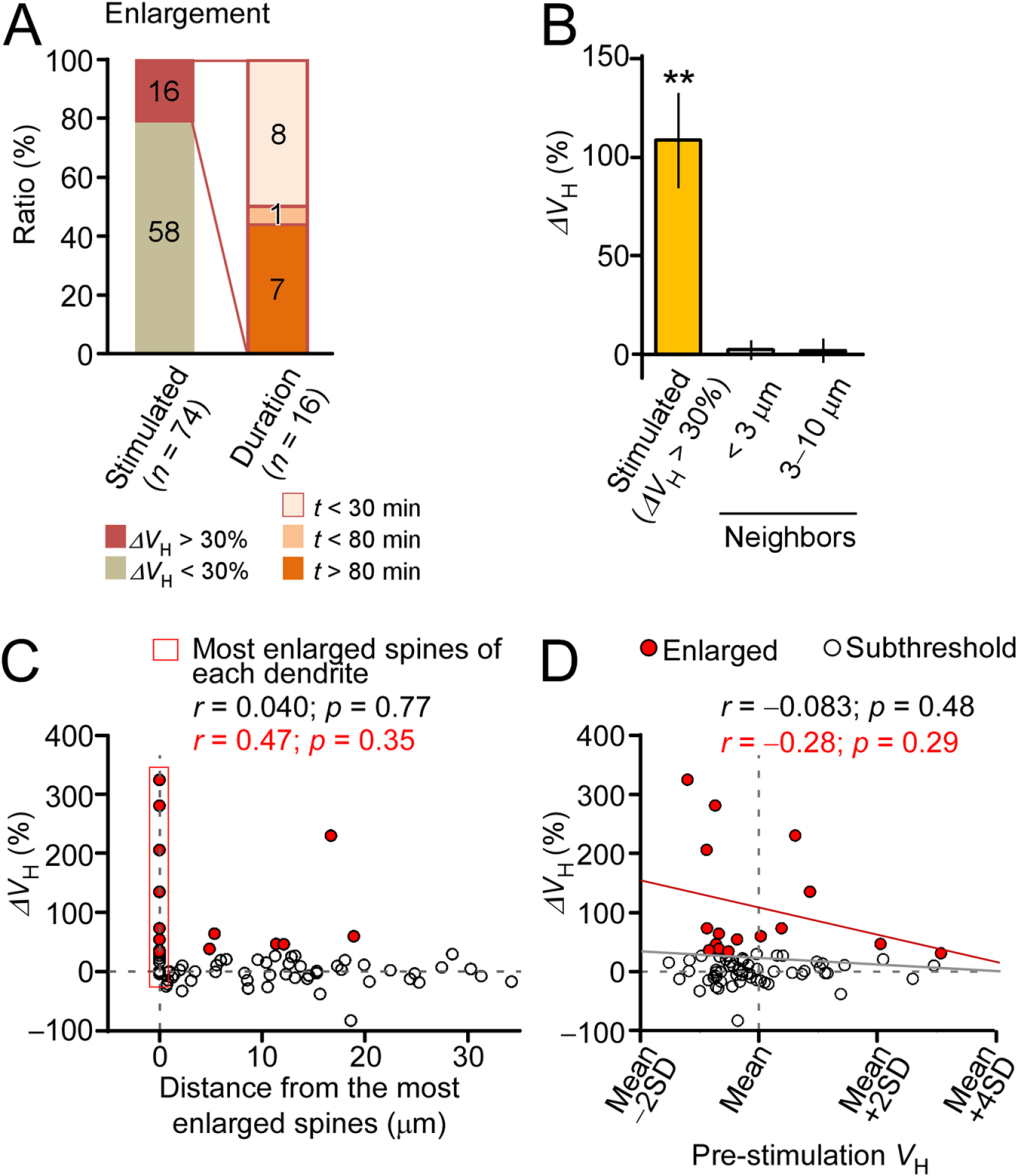
Properties of spine enlargement *in vivo*. (**A**) Ratio of head volume (ΔV_H_) change >30% in enlarged spines and enlargement durability. The left stacked bar represents the ratio of enlarged (22%) to remaining spines (78%) across all examined dendrites (20). The right stacked bar represents the distribution of enlargement durations. Numbers in the histograms indicate the number of spines. (**B**) The average increase in spine volume (109% ± 24%) across 16 stimulated spines (median = 62%, interquartile range [IQR] = 45%:153%) and neighboring spines located <3 µm away from stimulation site (2.2% ± 4.6%; median = 1.9%, IQR = −10%:21%; 15 spines) and 3–10 µm away from stimulation site(2.0% ± 6.0%; median = 6.0%, IQR = −4.4%:12%; 8 spines). **p < 0.01, based on Wilcoxon signed-rank test (versus zero). Error bars represent standard error of the mean. (**C**) A scatter plot of average spine enlargement (10–30 min after stimulation [i.e., ΔV_H_]) among stimulated spines compared to the distance between the most enlarged spines and other stimulated spines on each dendrite. (**D**) A scatter plot depicting average spine enlargement among stimulated spines compared to relative pre-stimulation spine head volume. Enlarged spines (ΔV_H_ >30%) are indicated by red circles. Pearson’s product-moment correlation coefficients and linear regression lines for all samples (gray) or for enlarged spines (red) are calculated for each scatter plot.

Spine enlargement was induced by two-photon glutamate uncaging, which was repeated 60 times at 1 Hz in close proximity to the spine heads (Fig. 1B,C). Volume changes varied among individual spines; however, the averaged time course revealed a transient increment phase followed by a stable plateau phase (Fig. 1D). For spines with >30% enlargement, the peak enlargement (10–30 min) and sustained phase of enlargement (>60 min) were 109% ± 24% (the mean ± the standard error of the mean: 16 spines/10 dendrites/10 mice) and 61% ± 20%, respectively. These values were significantly different (Wilcoxon test; p = 0.028) and ranged similarly but less than those of CA1 pyramidal neurons (203% ± 37% and 75% ± 20%) in slices^8^. Enlargements lasting more than 30 min occurred in eight of 16 enlarged spines (Fig. 2A) and were confined to stimulated spines (Figs 1D and 2B). The onset of enlargement was so rapid that volume increments were significant even at the first recording time point following uncaging (2 min vs. −30–0 min; by Wilcoxon signed rank test; p = 0.0016). The time-to-peak of the enlargement (~10 min) was longer than that in young hippocampal slices (<1 min). However, there was no significant difference among the amplitudes of enlargement at the three-time points after uncaging (2, 6 and 10 min; by two-way ANOVA [p = 0.36]). Thus, spine enlargement in the neocortex *in vivo* exhibited a rapid and transient enlargement, similar to what occurred in hippocampal slices^8^.

Enlargement was recorded in a small fraction of spines (22% of 74 spines/20 dendrites/18 mice; Fig. 2A) as compared to the fraction recorded in the hippocampal slices (approximately 95%)^8^. In spines without enlargement (ΔV_H_ <30%), average enlargement was negligible (−0.6% ± 2.5%) (Fig. 1D). Less frequent spine enlargement did not seem to be due to technical reasons, as enlargement was induced mostly in one spine (0–4 spines; average, 0.8 spine) of those that were simultaneously stimulated (1–7 spines; average, 3.7 spines), unlike in slices^8^. This conclusion was further supported quantitatively by the observation that the enlargement amplitudes of stimulated spines were no correlated with the distance of the spine from another spine that exhibited significant enlargement (Fig. 2C), despite only enlarged spines (ΔV_H_ >30%) being selected. We selected small spines (Fig. 2D) in which enlargement would be most pronounced in slice culture^8^. Enlargements were not correlated with spine depth or mouse age (Supplementary Fig. S2A,B), though only enlarged spines (ΔV_H_ >30%) were selected.

### Spine shrinkage *in vivo*

**Figure 3:**
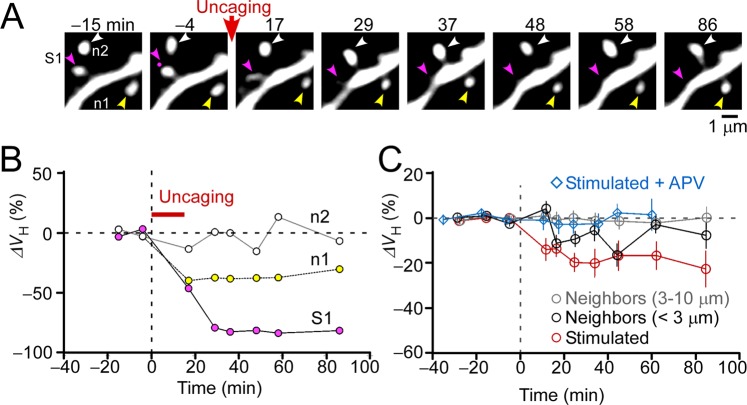
Induction of spine shrinkage *in viv*o. (**A**) Representative images of spine shrinkage. We stimulated, on average, 2.8 spines per dendrite using the same method as was used for enlargement but with a perfusion solution containing 1 mM magnesium (Mg^2+^). Spines (S1, magenta arrowheads) stimulated with low-frequency two-photon glutamate uncaging (1 Hz, 15 min) exhibited significant shrinkage. Some neighboring spines also shrunk (e.g., n1, yellow arrowheads), while others did not (e.g., n2, white arrowheads). The uncaging point is indicated by a small magenta dot. (**B**) Time-courses for spine head volume changes in panel (A). The magenta, yellow, and white circles indicate spine S1, n1, and n2 traces, respectively. (**C**) Average time courses for stimulated spines without (red circles) or with the NMDA receptor antagonist APV (blue diamonds). Forty-three spines were not exposed to APV while 12 were. Average time courses for spine neighbors located <3 μm (black circle) or 3–10 μm (gray circle) from the stimulated spines are also plotted (56 spines for <3 μm and 59 spines for 3–10 μm).

**Figure 4:**
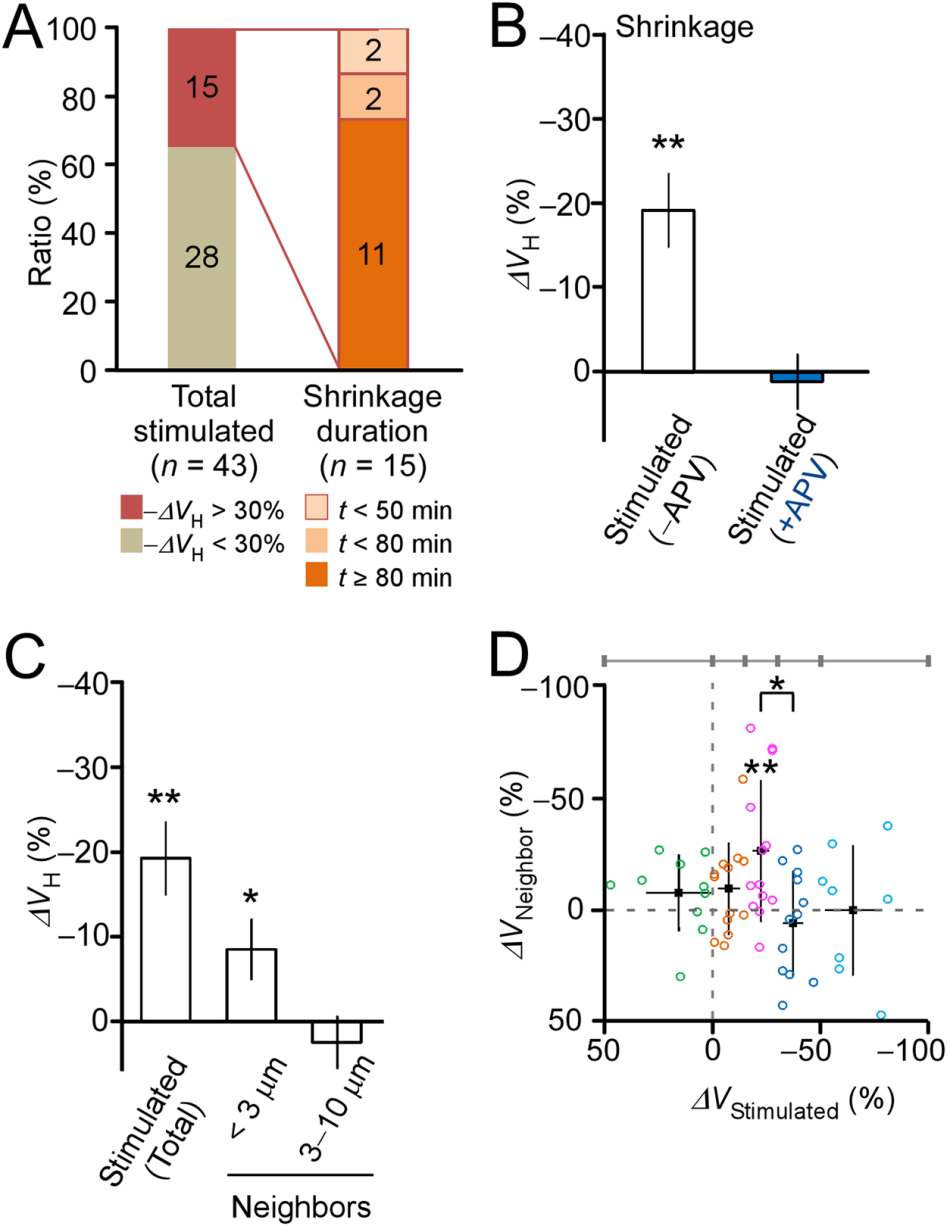
Properties of spine shrinkage *in vivo*. (**A**) The ratio of head volume shrinkage (−ΔV_H_) >30% among stimulated spines and shrinkage longevity. The left stacked bar chart depicts the ratio of shrunken spines to remaining spines for all dendrites (17). The right chart depicts the distribution of shrinkage durations. Numbers in the bars indicate the number of spines. (**B**) The bar graph depicts spine shrinkage (i.e., changes in head volume [ΔV_H_]) in the absence (−19% ± 4%; median = −18%, IQR = −37%:−1.5%; 43 spines) and in the presence (1.1% ± 3.3%; median = 2.0%, IQR = −5.8%:6.4%; 12 spines) of the NMDA receptor antagonist APV. Error bars represent the standard error of the mean (SEM). **p < 0.00014, based on Wilcoxon signed-rank test (versus zero). (**C**) The average amplitude of shrinkage among stimulated spines and their neighboring spines <3 µm away (−8.5% ± 3.6%; median = −9.6%, IQR = −22.0%:5.5%; 56 spines) or 3–10 µm (2.5% ± 3.1%; median = 2.5%, IQR = −10.4%:14.2%; 59 spines) from stimulated spines. *p < 0.05 and **p < 0.01, based on Wilcoxon signed-rank test (versus zero). The error bars represent SEM. (**D**) Spine shrinkage among neighboring spines (ΔV_Neighbors_) at <3 µm is plotted against spine shrinkage of stimulated spines (ΔV_Stimulated_). The average values are calculated within the ranges of ΔV_Stimulated_, as indicated above the plot and in Supplementary Fig. S5B. Samples within each range are indicated by color codes. Error bars represent standard deviation. Spine shrinkage spread is analyzed via a one-way ANOVA (p < 0.05). *p < 0.05, based on Tukey’s post hoc multiple comparison tests; **p < 0.01, based on Wilcoxon signed-rank test (compared to zero).

A solution containing a physiological concentration (1 mM) of Mg^2+^ was used to induce spine shrinkage^27^. Several spines on a dendrite were simultaneously stimulated with low-frequency two-photon glutamate uncaging (2.8 spines/dendrite average, 1–2 Hz for 10–15 min) (Fig. 3A). Stimulated spines exhibited as large of volume reductions (Fig. 3A,B, spine “S1”) over a gradual time course (Fig. 3C) as hippocampal slices^11,27^. We found that 35% of stimulated spines shrunk (−ΔV_H_ >30%, 15 of 43 spines/17 dendrites/8 mice) and that the mean amplitude at 20–50 min was 19% ± 4% (*n* = 43, median = 17.5, interquartile range = 1.5:37.4), similar to that found in the young hippocampal slices (23% ± 7%, *n* = 8)^7^. Furthermore, the shrinkage was persistent (>80 min) in most (73%) spines (Fig. 4A) and was absent when the NMDA receptor antagonist APV was added to the perfusion solution (Figs 3C and 4B).

Spine shrinkage spread to neighboring spines, which also occurred in hippocampal slice culture samples^11,27^. We calculated an average spine volume of the stimulated spines and neighboring spines 20–50 min from the onset of stimulation (Fig. 4C) and found that the spread of spine shrinkage was only significant in spines proximal to (<3 μm) those stimulated. Only 13% of spines within 3 μm of a stimulated spine exhibited shrinkage (−ΔV_Stimulated_ >30%; Fig. 4D). Interestingly, spines with greater shrinkage tended to display less spread (Fig. 4D). Thus, the spread of spine shrinkage was less frequent in the adult neocortex *in vivo* than in young hippocampal slices in which shrinkage spread to 71% of spines within 3 µm and to 38% of spines within 7 µm of a stimulated spine^11,27^.

We found that the prestimulation spine volume was weakly and non-significantly correlated with spine shrinkage (Supplementary Fig. S3A). Spine retraction also occurred during spine shrinkage (Supplementary Fig. S3B,C)^11^; however, spine shrinkage was non-significantly correlated with retraction (ΔSpine length; Supplementary Fig. S3C). We did not observe any interspine distance dependency on the induction of spine shrinkage (Supplementary Fig. S4A). Spine shrinkage was also insignificantly correlated with dendritic depth (Supplementary Fig. S4B).

## Discussion

In the current study, we present evidence for the successful induction of spine enlargement and shrinkage by uncaging glutamate in the adult mouse neocortex *in vivo*^28^. The essential features of this plasticity were similar to those reported previously in the hippocampal slices^8^. For enlargement, a rapid transient phase and sustained enlargement was noted, which was confined to stimulated spines. Shrinkage, however, occurred gradually and spread to neighboring spines.

A major difference between *in vivo* adult mouse neocortex changes here and those that occurred in hippocampal slice preparations previously^8^ was the success rate for the induction of spine enlargements (22% and 95%, respectively). This may have been due to differences in tissue age or other factors between the neocortex *in vivo* and the hippocampal slices. Additionally, difficulty in controlling concentrations of Mg^2+^ ions, the usage of anesthesia, types of neurons, parts of dendrites, and other technical reasons may have led to differences between the two paradigms. Importantly, once enlargement was induced, amplitude and persistence were similar between cortex and young hippocampal preparations. Moreover, the diversity cannot be simply explained by technical issues, as even one stimulated spine showed enlargement, however, other simulatneously stimulated neighboring spines did not, despite exposed to the same uncaging stimuli (Fig. 2C). Critically, we were able to determine that glutamate uncaging did in fact occur, as the induction of shrinkage was similar in hippocampal slice preparations (34–38%). These suggest some spine enlargement heterogeneity among neocortical spines *in vivo*.

As seen in the hippocampal slices, the spread of spine shrinkage to neighboring spines was also found in the neocortex^11^. However, neighboring spine shrinkage occurred less in neocortex than in young hippocampus. It should be noted, however, that shrinkage spread was dependent on the stimulation protocol, even in the same preparations. Spread was also more pronounced during spike-timing dependent plasticity (STDP)^11^ but negligible when low frequency (0.1 Hz) uncaging was paired with a 200 ms depolarization^12^. Thus, it is possible that such spread may be more extensive *in vivo* during STDP, which may help competition for neighboring plasticity^11^ and the removal of clustered spines^29–34^.

Thus, the two-photon *in vivo* uncaging technique used here led to a quantitative difference in the structural plasticity of dendritic spines in the adult neocortex *in vivo* as compared to that which occurred in hippocampal slice culture preparations. This supports the notion that the cortex is slower to learn than the hippocampus^35^. Although the expression of synaptic molecules is highly variable from spine to spine^36^, the molecular basis of this heterogeneity in enlargement requires further investigation.

## Methods

### Surgical procedures

All animal procedures were approved by the Animal Experiment Committee of the University of Tokyo (Tokyo, Japan). Procedures were conducted in accordance with the University of Tokyo Animal Care and Use Guidelines. Surgical procedures were performed as described previously^7^. In brief, we anesthetized adult mice expressing YFP or GFP in a subset of neurons: Thy1 YFP in the H line [YFP-H] or GFP in the M line [GFP-M]. Eighteen mice, aged 148 ± 129 days (mean ± the SD), were used for enlargement experiments (17 YFP-H mice; one GFP-M mouse) (Supplementary Fig. S2B). Eight mice, aged 70 ± 19 days, were used for shrinkage experiments (five YFP-H mice; three GFP-M mice) (Supplementary Fig. S4C). Mice were anesthetized with intraperitoneal injections of urethane and xylazine at 1.2 g/kg body weight and 7.5 mg/kg body weight, respectively, which were supplemented with subcutaneous administration of the analgesic ketoprofen (20 mg/kg body weight). A steel plate with a recording chamber was attached to the skull with cyanoacrylate glue such that the recording chamber was positioned just above the visual cortex (3.0 mm posterior, 2.5 mm lateral to the bregma)^37^. The plate was then tightly fixed to the metal platform. We then removed the skull using a pair of forceps and a dental drill, which was fixed to a stereotaxic instrument (Narishige, Tokyo, Japan). The dura mater was carefully removed using fine forceps and a microhook to minimize any pressure applied to the surface of the brain. We then placed a semicircular glass coverslip to cover approximately one-half of the exposed brain surface (Fig. 1A). The coverslip was fixed using either dental acrylic (Fuji-Lute BC; GC Corp., Tokyo, Japan) or a stainless steel wire. Mice were supplied with humidified oxygen gas and warmed to 37 °C with a heating pad (FST-HPS; Fine Science Tools Inc., North Vancouver, Canada) during all surgical procedures.

### Two-photon *in vivo* imaging and uncaging

*In vivo* two-photon imaging and uncaging were conducted using an upright microscope (BX61WI; Olympus, Tokyo, Japan) equipped with a FV1000 laser scanning microscope system (Olympus) and a water-immersion objective lens (LUMPlanFI/IR 60X with a numerical aperture of 0.9; Olympus). The system included two mode-locked femtosecond-pulse titanium-sapphire lasers (MaiTai; Spectra Physics, Mountain View, CA, USA), one set to 720 nm for uncaging^1^ and the other to 980 nm for imaging. Each light path was connected to the microscope via an independent scan head and acousto-optic modulator. For 3-D reconstruction of dendrite images, 21–40 XY images separated by 0.5 μm were stacked by summing fluorescence values at each pixel. 4-Methoxy-7-nitroindolinyl (MNI)-glutamate or 4-carboxymethoxy-5,7-dinitroindolinyl (CDNI)-glutamate was custom-synthesized by the Nard Institute Ltd. (Amagasaki, Japan) or purchased from Tocris Bioscience (Bristol, UK) and perfused through the recording chamber via artificial cerebral spinal fluid (ACSF).

### *In vivo* enlargement of dendritic spines

For *in vivo* spine enlargement experiments, the cortical surface was first superfused with magnesium-free ACSF (ACSF without Mg^2+^) containing 125 mM NaCl, 2.5 mM KCl, 3 mM CaCl_2_, 0 mM MgCl_2_, 1.25 mM NaH_2_PO_4_, 26 mM NaHCO_3_, 20 mM glucose, and 10 µM tetrodotoxin (Nacalai, Kyoto, Japan). This solution was bubbled with 95% oxygen and 5% carbon dioxide for approximately 30 ± 15 min (mean ± the SD; 20 dendrites). The bathing solution was then changed to ACSF without Mg^2+^ containing 20 mM MNI-glutamate or 10 mM CDNI-glutamate and 200 μM Trolox (Sigma-Aldrich, St. Louis, MO, USA), which diffused into the cortical extracellular space approximately 15 min before the uncaging experiments. Two-photon uncaging was aimed at the tip of the spines and repeated 60 times at 1 Hz. The power of the uncaging laser was typically set to 10 mW for 0.6 ms. We expected that transient currents similar to miniature excitatory-postsynaptic currents were elicited approximately at this laser power; however, we did not change power levels according to cortical depth^7^.

For each experiment, 2–8 spines (average, 4.6 spines) were stimulated along a dendrite. We studied 52 spines/15 dendrites/14 mice with MNI-glutamate and 22 spines/5 dendrites/4 mice with CDNI-glutamate. The enlargement success rates were 25% and 13%, respectively. The solution was pooled in a small reservoir (2 mL) (Fig. 1A). Pure water was constantly added (after empirically determining its flow rate) to the reservoir to maintain an osmotic pressure in the solution of approximately 320 mOsm/kg. The solution was warmed to 37 °C in the chamber with circulating hot water (Fig. 1A). All physiological experiments were conducted at 37 °C.

### *In vivo* shrinkage of dendritic spines

For all spine shrinkage experiments, the cortical surface was superfused with ACSF containing 2 mM CaCl_2_ and 1 mM MgCl_2_. The solution was then changed before uncaging experiments to ACSF, which contained 200 μM of Trolox and a caged compound (i.e., 20 mM MNI-glutamate or 10 mM CDNI-glutamate). We studied 38 spines/15 dendrites/7 mice with MNI-glutamate and 5 spines/2 dendrites/1 mice with CDNI-glutamate. The success rate of shrinkage was 37% and 25%, for MNI-glutamate and CDNI-glutamate, respectively. Repetitive stimulation was conducted at 1–2 Hz for 10–15 min at a laser power similar to that used for enlargement (~10 mW).

As a control, stimulation was delivered in the presence of 50 mM d-2-amino-5-phosphonovaleric acid (APV), an NMDA receptor antagonist with MNI-glutamate.

### Spine volume analyses

Spine head volumes were estimated from the total fluorescence intensity of spines by summing the fluorescence values of stacked images in 3-D using Image-J software (NIH, Bethesda, Maryland, USA), as reported previously^7^. When images contained axon fibers that overlapped with target dendrites at different image depths, the spine head volume in the dendrite was calculated by partially summing the fluorescence values of five sequential Z-images by taking the moving average of the image stack along the Z-plane. This was done to avoid axonal fibers. Because dendritic spines are near the diffraction limit of a two-photon microscope, partially summed values (2-μm range in the Z-direction) were used to reflect spine volumes. Thus, the maximum value of Z-moving average images allowed for good approximation of total Z-summed stacked images.

Dendritic spines exhibit spontaneous fluctuations in fluorescence because of spontaneous morphological changes, motility, and measurement errors^13^. To determine spine volume fluctuations, we calculated the CV of *in vivo* images before glutamate uncaging (14.7% ± 16.1% for 227 spines in the enlargement condition and 12.5% ± 7.9% for 196 spines in the shrinkage condition). We set fluctuation limit values with a baseline of two CVs (i.e., 30% for enlargement data; 25% for shrinkage data) and discarded data when fluctuations exceeded this limit. Stimulated and neighboring spines with prestimulation fluctuations over this limit were discarded due to instability. For spine volume analyses, average spine volumes during the 10–30 min (for enlargement) and 20–50 min (for shrinkage) following stimulation were calculated and to indicate any differences from the baseline volume.

### Spine length analysis

To determine spine length before and after stimulation, the length between the tip of the spine and the edge of the dendrite of interest was measured on Z-stack images (Supplementary Fig. S3B).

### Statistical analysis

All data are presented as mean ± standard error of the mean (*n* indicates the number of spines), unless otherwise stated. Statistical tests of spine outcomes were conducted using Excel-Statistics software (Social Survey Research Information Co. Ltd., Tokyo, Japan). Differences from baseline values or between values were analyzed using the Wilcoxon signed-rank test (Figs 1D, 2B, 4B–D and S3B). Delays in the enlargement peak were analyzed via a two-way ANOVA (Fig. 1D). Spread of spine shrinkage was analyzed via a one-way ANOVA, followed by Tukey’s post hoc multiple comparison testing (Fig. 4D). Pearson’s product-moment correlation coefficients were calculated for scatter plots (Figs 2C,D, S2A,B, S3A,B and S4A–C). The significance of a correlation coefficient was determined via *t*-test. Differences between mean values for the two groups were analyzed using the Mann-Whitney rank-sum test (Fig. S5A).

## Supplementary information

Supplemetary Figures
