# Supplementary material for: Bidirectional in vivo structural dendritic spine plasticity revealed by two-photon glutamate uncaging in the mouse neocortex
Source: Sci Rep. 2019 Sep 26;9:13922. doi: 10.1038/s41598-019-50445-0 (PMC6763442; doi:10.1038/s41598-019-50445-0)
Supplement: Supplementary file 1 — Supplemetary Figures [file 41598_2019_50445_MOESM1_ESM.pdf]

Supplementary Information

Bidirectional *in vivo* structural dendritic spine plasticity revealed by two-photon glutamate uncaging in the mouse neocortex

Jun Noguchi, Akira Nagaoka, Tatsuya Hayama, Hasan Ucar,
Sho Yagishita, Noriko Takahashi & Haruo Kasai

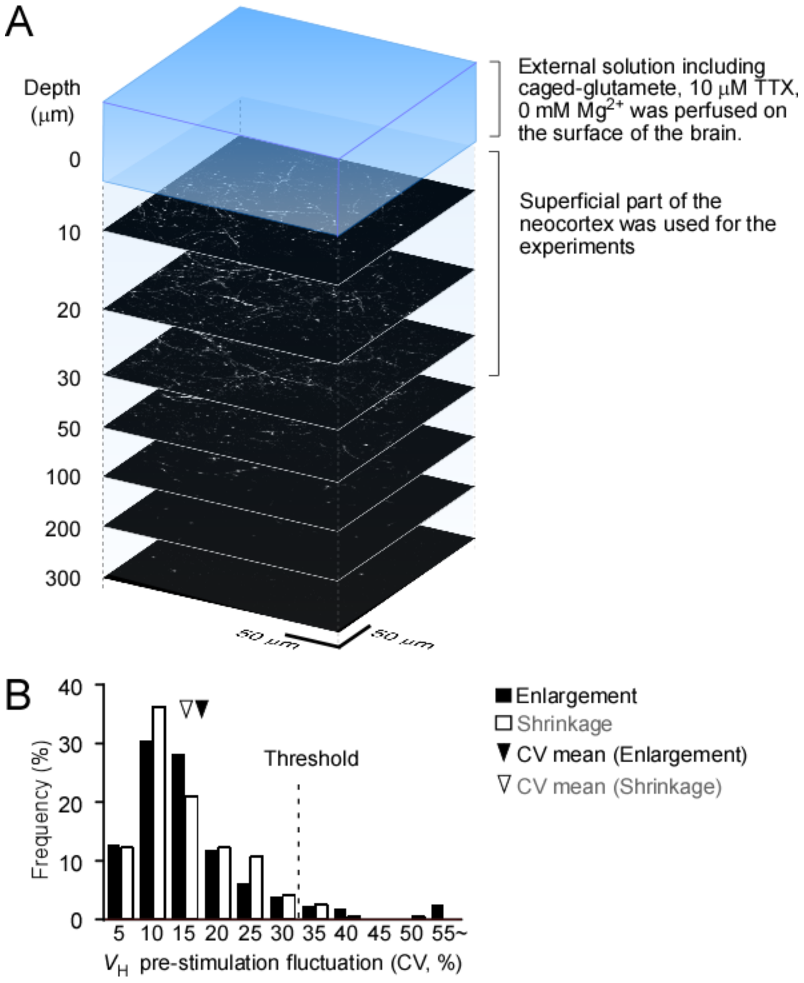

Supplementary Figure S1. A diagram of the typical *in vivo* uncaging experiment.

(**A**) The surface of the cortex is superfused with artificial cerebral spinal fluid (ACSF) solution containing 4-carboxymethoxy-5,7- dinitroindolinyl-glutamate (CDNI-Glu) but devoid of magnesium (Mg^{2+}). (**B**) The amplitude histogram of prestimulation spine volume fluctuations. The mean coefficient of variation (CV) is approximately 15% and is unaltered by the low Mg^{2+} solution. We thus set the threshold at 30% (i.e., 2 CV) for enlargement and for shrinkage.

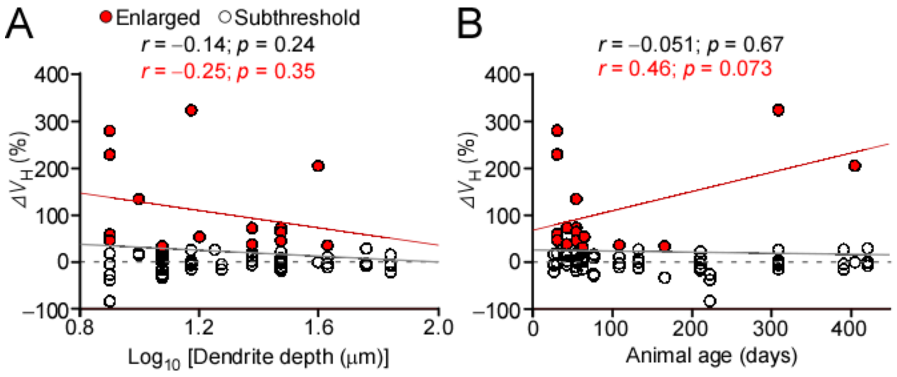

Supplementary Figure S2. Investigation of the spine enlargement conditions.

Scatter plots of the average spine enlargement of the stimulated spines (i.e., change in the head volume [ΔV_H]) at 10–30 min after the stimulation against (**A**) the common log of the dendrite depth, (**B**) and age of mice. Average enlargement of the stimulated spines from animals 0–60 days old (42.3% ± 14.2%, 26 spines, 5 mice), 61–200 days old (8.9% ± 4.7%, 23 spines, 6 mice), and 200+ days old (16.6% ± 16.0%, 25 spines, 7 mice). The enlarged spines (ΔV_H >30%) are marked by red circles. Pearson's product-moment correlation coefficients and linear regression lines for all samples (gray) or for the enlarged spines (red) are calculated for the scatter plots.

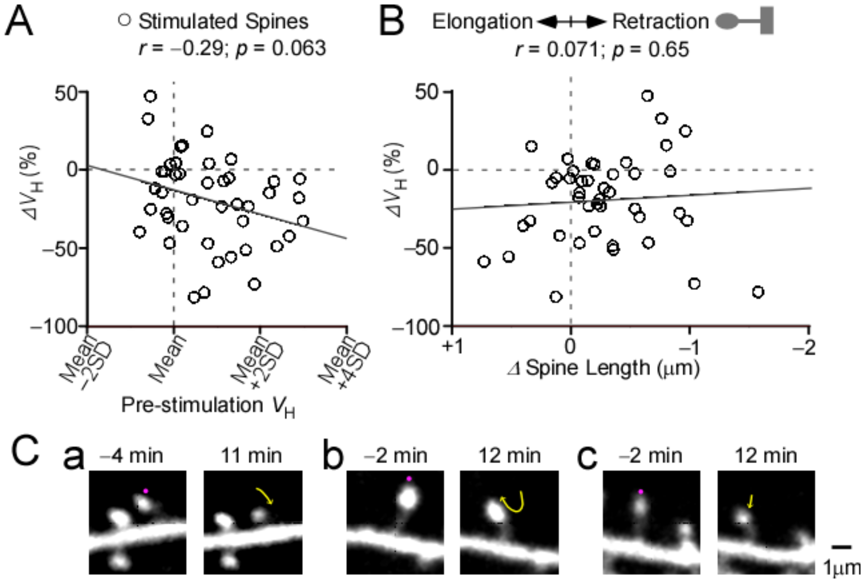

Supplementary Figure S3. Properties of spine shrinkage. (**A**,**B**) The scatter plots of the average spine shrinkage (ΔV_H) of the stimulated spines against the spine properties present (**A**) the relative prestimulation spine head volume in each dendrite and (**B**) the spine retraction just after the end of the stimulation. The average retraction was −0.28 ± 0.07 μm (43 spines) and was significant ($p = 0.0004$), based on Wilcoxon signed-rank test against zero. (**C**) Three representative images of spine retraction after the low-frequency stimulation. The magenta dots and yellow arrows represent the uncaging points and direction of the retraction, respectively.

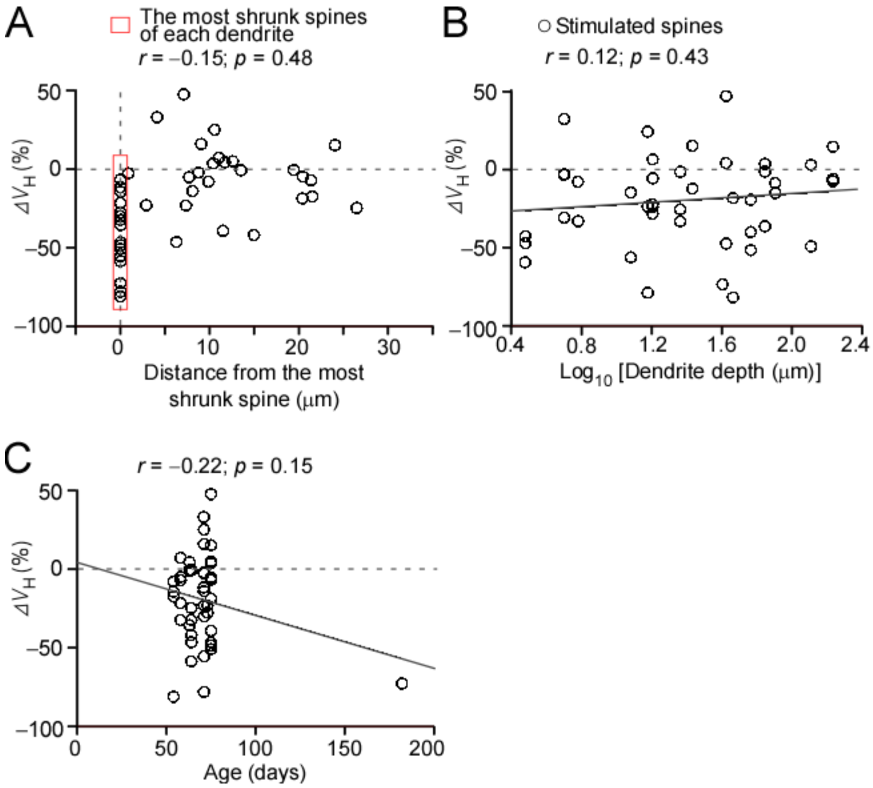

Supplementary Figure S4. Conditions of spine shrinkage. Scatter plots of the average spine shrinkage (i.e., change in the head volume [ΔV_H]) of the stimulated spines against (**A**) the distance between the most shrunken spine of each dendrite and other stimulated spines, (**B**) the common logarithm of the dendrite depth, and (**C**) the animal's age.

A

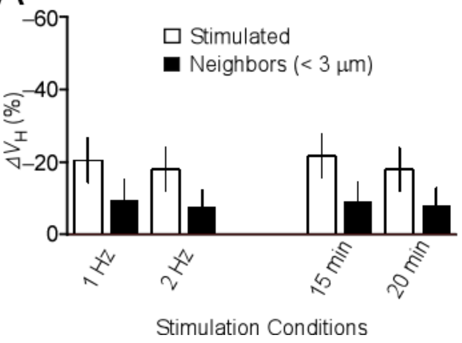

B

$\Delta V_{Stim.}$ Bin (%)	+50 ~ 0	0 ~ −15	−15 ~ −30	−30 ~ −50	−50 ~ −100
n	10	13	13	12	8
$\Delta V_{Neighbor}$ Mean	−7.7	−9.6	−26.6	+5.9	+0.1
$\Delta V_{Neighbor}$ Median	−10.9	−15.0	−11.5	+3.1	−6.8

Supplementary Figure S5. Dependence of spine shrinkage and its spread on the frequency and duration of stimulation. (**A**) Amplitudes of spine shrinkage of the stimulated (open bars) and neighboring spines (filled bars) plotted against the frequency of stimulation (1 or 2 Hz) and the duration (15 or 20 min). The average spine shrinkage ($\Delta V_{Stimulated}$) was not significantly different between 1Hz (−20.4% ± 6.2%, 23 spines) and 2Hz (−17.9% ± 6.1%, 20 spines) nor between 10 min (−21.6% ± 6.0%, 16 spines) and 15 min (−17.8% ± 6.0%, 27 spines) (p = 0.86 and 0.67, respectively based on Mann-Whitney rank-sum test). The average shrinkage spread (< 3 μm)($\Delta V_{Neighbor}$) was not also significantly different between 1Hz (−9.6% ± 5.7%, 26 spines) and 2Hz (−7.6% ± 4.6%, 30 spines) nor between 10 min (−9.2% ± 5.3%, 25 spines) and 15 min (−7.9% ± 5.0%, 31 spines) (p = 0.69 and 0.93, respectively based on Mann-Whitney rank-sum test). (**B**) A table showing the bin range of the shrinkage spread analysis (related to Fig. 4D). In addition to the bin range, the sample numbers, mean and median $\Delta V_{Neighbor}$ of each bin are shown. Tukey's post hoc multiple comparison tests represented that the difference of the shrinkage spread was only significant between 15-30% bin and 30-50% bin (p = 0.017) but not between other combinations such as between 15-30% bin and 50-100% bin (p = 0.14), or between 0-15% bin and 30-50% bin (p = 0.53).
